# Effect of Rehabilitation with Extremely Low Frequency Electromagnetic Field on Molecular Mechanism of Apoptosis in Post-Stroke Patients

**DOI:** 10.3390/brainsci10050266

**Published:** 2020-04-30

**Authors:** Natalia Cichon, Ewelina Synowiec, Elzbieta Miller, Tomasz Sliwinski, Michal Ceremuga, Joanna Saluk-Bijak, Michal Bijak

**Affiliations:** 1 Biohazard Prevention Centre, Faculty of Biology and Environmental Protection, University of Lodz, Pomorska 141/143, 90-236 Lodz, Poland; natalia.cichon@biol.uni.lodz.pl; 2 Department of Molecular Genetics, Laboratory of Medical Genetics, Faculty of Biology and Environmental Protection, University of Lodz, Pomorska 141/143, 90-236 Lodz, Poland; ewelina.synowiec@biol.uni.lodz.pl (E.S.); tomasz.sliwinski@biol.uni.lodz.pl (T.S.); 3 Department of Neurological Rehabilitation, Medical University of Lodz, Milionowa 14, 93-113 Lodz, Poland; elzbieta.dorota.miller@umed.lodz.pl; 4 Military Institute of Armament Technology, Prymasa Stefana Wyszyńskiego 7, 05-220 Zielonka, Poland; ceremugam@witu.mil.pl; 5 Department of General Biochemistry, Faculty of Biology and Environmental Protection, University of Lodz, Pomorska 141/143, 90-236 Lodz, Poland; joanna.saluk@biol.uni.lodz.pl

**Keywords:** extremely low frequency electromagnetic field, apoptosis, neuroplasticity, stroke

## Abstract

Apoptosis in acute stroke is associated with a negative prognosis and is correlated with the severity of the neurological deficit. However, there is no evidence that indicates that, in the subacute phase of the stroke, the apoptosis process might activate neuroplasticity. Therefore, in this study, we investigated the effect of an extremely low frequency electromagnetic field (ELF-EMF) on the molecular mechanism of apoptosis, as used in the rehabilitation of post-stroke patients. Patients with moderate stroke severity (*n* = 48), 3–4 weeks after incident, were enrolled in the analysis and divided into ELF-EMF and non-ELF-EMF group. The rehabilitation program in both groups involves the following: kinesiotherapy—30 min; psychological therapy—15 min; and neurophysiological routines—60 min. Additionally, the ELF-EMF group was exposed to an ELF-EMF (40 Hz, 5 mT). In order to assess the apoptosis gene expression level, we measured the mRNA expression of *BAX*, *BCL*-*2*, *CASP8*, *TNFα*, and *TP53*. We found that ELF-EMF significantly increased the expression of *BAX*, *CASP8*, *TNFα*, and *TP53*, whereas the *BCL-2* mRNA expression after ELF-EMF exposition remained at a comparable level in both groups. Thus, we suggest that increasing the expression of pro-apoptotic genes in post-stroke patients promotes the activation of signaling pathways involved in brain plasticity processes. However, further research is needed to clarify this process.

## 1. Introduction

The symptoms of brain damage caused by cerebral ischaemia are the consequence of massive cell death in the ischaemic area. Ischaemic cascades—successive biochemical changes leading to degradation of cell structures and membranes, and ultimately to brain cell death—activated after a few seconds of blockage of the cerebral blood flow [1]. Ischaemia causes cell death as a result of necrosis, apoptosis, and autophagy, the markers of which have been well documented [2,3,4,5].

There are many factors that can induce apoptosis of cells after ischaemia, such as inflammation, cytokine activation, cascade of free radicals, and induction of thrombin [6]. Neuronal apoptosis is regulated by various genes, such as *BCL-2* (inhibitor of apoptosis) and *BAX* (activator of apoptosis) [7]. The BCL-2 protein (B cell lymphoma) is a product of the *BCL-2* gene located on chromosome 18 at the 18q21.3 locus. Under normal conditions, the expression of *BCL-2* is regulated by the p53 protein, and its main function is to protect the cell from apoptosis [8]. The p53 protein is called the ‘genome guard’, and plays an important role in the regulation of apoptosis as a transcription factor [8]. Increased expression of *BCL-2* causes production of the BCL-2/BAX heterodimer and inhibition of cell apoptosis, whereas with an increased expression of *BAX*, the BAX/BAX homodimer is produced and cell apoptosis is activated. When *BCL-2* is elevated and *BAX* is reduced, the BCL-2/BAX ratio increases and the cell might survive [9]. Caspases are evolutionary, conserved proteins, which play a key role in the mechanism of apoptosis and inflammation. The role of initiator caspases with a long pro-domain, including caspase 8, is in signal detection and activation of the cascade, leading to apoptosis [10].

Post-stroke recovery is associated with the compensatory plasticity of the brain, and is activated by rehabilitation. There are data showing that, in the subacute phase of stroke, proapoptotic pathways and mechanisms of neuroplasticity may be interrelated [11,12]. After a stroke, neuroplasticity begins immediately after the ischemic incident. Neuroplasticity involves synaptic potentiation and formation of a new synaptic junction. Although they remain in a weakened state, the connection between the brain centres is activated, and any damage to its function can be reintroduced partly or comprehensively, because the function of the damaged area has been assumed by other subcortical or cortical structures [13]. Neuroplasticity is associated with neurogenesis, in which fully functional nerve cells are produced. Nerve cells are generated essentially in the subgranular zone (SGZ) and the sub-ventricular zone (SVZ) [14]. Microenvironmental factors, growth factors, neurotrophins, hormones, and neurotransmitters regulate this process [15].

Post-stroke rehabilitation is a complex process, consisting of various therapeutic treatments. Correctly conducted rehabilitation is one of the most important forms of therapy after stroke. Low frequency electromagnetic field (ELF-EMF) therapy is one of the methods used in rehabilitation after stroke. This type of treatment is characterized by physical parameters describing ELF-EMF; that is, frequency (<50 Hz), magnetic induction (<10 mT), as well as the shape of the pulse (rectangular, trapezoidal, triangular, sinusoidal, unipolar, bipolar) [16]. ELF-EMF therapy has been noted for the minimal level of harm it causes, the extensive range of ways in which it can be used to treat various diseases, as well as the beneficial clinical results achieved with relatively small financial outlay. The nature of the ELF-EMF effect on the body allows deep tissue penetration, however, it is not a thermal method, which makes it possible to use it in the treatment of neurological diseases [17]. ELF-EMF affects the body at both the molecular and cellular level and causes the change in ionic channels. However, there is in fact no known mechanism in which calcium channels can be directly activated by ELF-EMF. This effect can be a consequence of mitochondrial and metabolic disturbances [18]. ELF-EMF therapy used in physical rehabilitation, including after strokes, increases muscle strength, reduces muscle spasticity as a consequence of pyramidal tracts damage, has an analgesic effect, as well as increases blood flow. Thereby, ELF-EMF affects regeneration of nerve tissue through improvements to its metabolism [19]. Despite the many substantiated beneficial effects of ELF-EMF, this form of therapy is not a routine method for post-stroke rehabilitation.

Apoptosis in stroke patients is an ambiguous process. On the one hand, increased apoptosis in the acute phase of a stroke is correlated with larger neurological deficits. On the other hand, the process of programmatic cell death is one of the physiological mechanisms for eliminating damaged cells [20]. Our previous research confirms the significant effect of ELF-EMF on the enhancement of neuroplasticity and biochemical parameters correlated with the improvement of physical and motor condition according to clinical scales (Mini-Mental State Examination—MMSE, Activities of Daily Living—ADL, Geriatric Depression Scale—GDS) [21,22,23,24,25]. In this work, we undertook assessment of the effect of ELF-EMF on the crucial mechanisms of apoptosis in order to search for the molecular basis of ELF-EMF activity.

## 2. Materials and Methods

### 2.1. Subjects Presentation and Blood Collection

Forty-eight patients with moderate stroke severity, 3–4 weeks after incident, were enrolled for the analysis. Twenty-six patients were recruited into the ELF-EMF study group and twenty two patients were recruited into the non-ELF-EMF control group. The NIHSS scores were 4.9 ± 3.1 and 5.4 ± 2.9 for the study group and control group, respectively; mean age was 48.8 ± 7.7 and 44.8 ± 8.0, respectively. Exclusion criteria included the following: chronic or significant acute inflammatory factors, dementia, haemorrhagic stroke, neurological illness other than stroke, and/or decreased consciousness in their medical pre-stroke history. Patients with metal and/or electronic implants were excluded from the study group. Neurorehabilitation with neurological and internal examination was conducted for four weeks at the Neurorehabilitation Ward of Dr K. Jonscher Municipal Medical Center in Lodz, Poland. This consisted of the following: kinesiotherapy—30 min; psychological therapy—15 min; and neurophysiological routines—60 min; as well as ELF-EMF treatment.

Blood samples were collected into citrate, phosphate, dextrose, adenine (CDPA1) containing tubes, before and after ten sessions of therapy, at the same time of day (between 07:00 and 09:00), under fasting conditions. They were frozen at −80 °C immediately upon collection. All blood samples were collected and stored using the same protocol. The study was conducted in accordance with the principles of the Helsinki Declaration. The Bioethics Committee of the Faculty of Biology and Environmental Protection of the University of Lodz, Poland, confirmed this study with Resolution No. 13/KBBN-UŁ/II/2016.

### 2.2. Setting and Treatment of ELF-EMF

**Figure 1:**
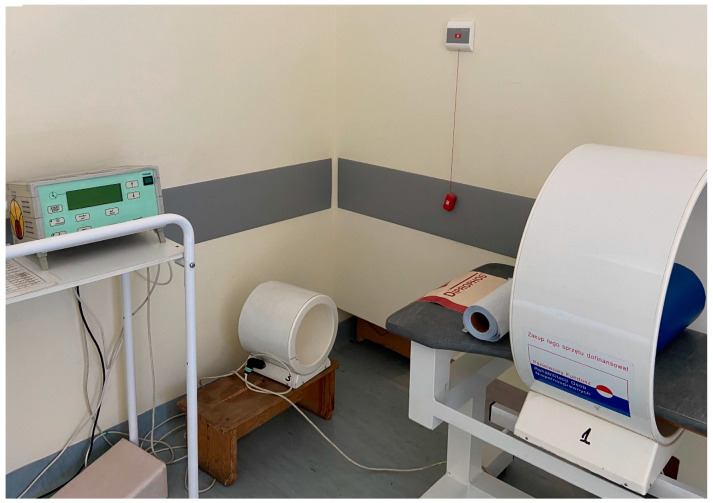
Device to generate extremely low frequency electromagnetic field (ELF-EMF)—Magnetronic MF10 (EiE Elektronika i Elektromedycyna, Otwock, Poland).

Magnetronic MF10 generator (EiE Elektronika i Elektromedycyna, Otwock, Poland) was used to generate ELF-EMF with the following parameters: magnetic induction—5 mT, frequency—40 Hz, wave forms—rectangular, bipolar, time of session—30 min, and exposed area—pelvic girdle (Figure 1). In this research, a coil applicator with five layers of 187 turns of 1.45 mm twin-parallel wires was used with the following parameters: length—270 mm and diameter—550 mm. ELF-EMF intensity inside the solenoid was nonequivalent. The induction coils of the generator were assembled horizontally, while apportionment of ELF-EMF was vertical. The established value of magnetic induction of 5 mT was located in the applicator geometrical center, whereas, with the proximity of the coil surface, the change of ELF-EMF intensity increased by around plus 1.4. The computer system controlled ELF-EMF itself. Post-stroke patients were exposed in 10 standard sessions, five times per week for two weeks. All subjects were placed in applicator, but only patients in the ELF-EMF group were exposed to ELF-EMF, and patients in the non-ELF-EMF group were given sham exposures. The sham exposure was obtained by setting, but not turning on the device.

### 2.3. Isolation of RNA

Reagent^®^ (Sigma-Aldrich, Saint Louis, MO, USA) was used for lysis of frozen samples, following which phase separation was effectuated. InviTrap Spin Universal RNA Mini Kit (Stratec Biomedical Systems, Birkenfeld, Germany) was used for purification of the RNA-containing aqueous phase. A Synergy HTX Multi-Mode Microplate Reader, equipped with a Take3 Micro-Volume Plate (BioTek Instruments, Inc., Winooski, VT, USA), was used to estimate, purify, and quantify the RNA.

### 2.4. Reverse Transcription

Diluted RNA samples (to 20 ng/µL) were transcribed onto cDNA using a High-Capacity cDNA Reverse Transcription Kit (Applied Biosystems™, Waltham, MA, USA). All procedures were conducted in accordance with the producents’ protocol.

### 2.5. Real-Time PCR

Levels of expression of the investigated genes (*BAX*, *BCL*-*2*, *CASP8*, *TNFα*, and *TP53*) were obtained using TaqMan probes: Hs00180269_m1 for human *BAX* gene; Hs00608023_m1 for human *BCL-2* gene; Hs01018151_m1 for human *CASP8* gene; Hs00174128_m1 for human *TNFα* gene; Hs01034249_m1 for human *TP53* gene, and Hs99999905_m1 for human *GAPDH* gene (endogenous control) (Life Technologies, Carlsbad, CA, USA). A TaqMan Universal Master Mix II (Life Technologies, Carlsbad, CA, USA) was used to perform the real-time PCRs, which were executed in a CFX96 real-time PCR system (BioRad Laboratories, Hercules, CA, USA). All steps were conducted according to the producers’ recommendations. The equation 2-ΔCt (ΔCt = Ctstudied gene - CtGAPDH) was used to calculate the relative expressions of the analyzed genes.

### 2.6. Data Analysis

All assays were conducted twice and calculated as mean values. For all participants, the values of the expression before treatment were used as the output value (100%). Data from the assays executed on these same participants after treatment were formulated as a percentage of the output value. Data obtained in this way were expressed as mean ± SD.

Stats Direct statistical software v.2.7.2 was used for all statistical analyses. Paired Student’s *t*-tests (for normal distribution) and Wilcoxon tests (for distribution deviating from normal) were used to analyze significant differences between the data obtained for subjects before and after treatments. Changes in parameters after appropriate treatments for comparison of differences between the ELF-EMF group and the non-ELF-EMF group were calculated. For this analysis, unpaired Student’s *t*-tests or Mann–Whitney *U* tests were used. Additionally, a correlation analysis between the changes in experimental and clinical parameters was executed using a Spearman’s rank correlation. A level of *p* < 0.05 was accepted as statistically significant for all analyses.

## 3. Results

**Figure 2:**
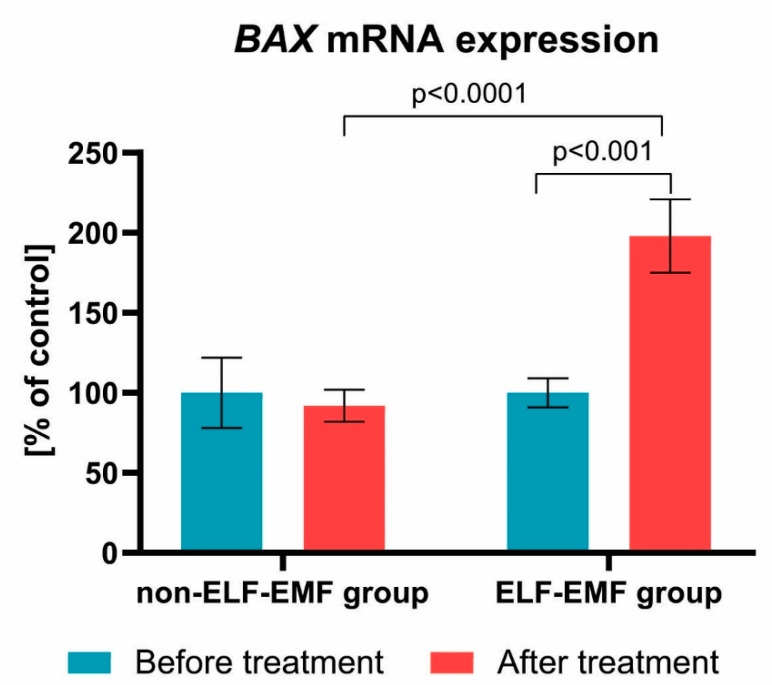
Comparison of the *BAX* mRNA expression acquired from the study group vs. control. Results formulated as parameter changes before and after therapy (100% expressed for the level of *BAX* mRNA expression in each patient sample before treatment). Statistical significance between ELF-EMF and non-ELF-EMF groups after 10 sessions amount to *p* < 0.0001.

Our comparative analysis shows the effect of ELF-EMF therapy on the expression level of various genes involved in apoptosis. As presented in Figure 2, the expression of the *BAX* gene in the ELF-EMF group after 10 sessions of rehabilitation was significantly higher as compared with the non-ELF-EMF group (*p* < 0.001). The increase of the *BAX* mRNA gene expression level in the ELF-EMF group was about 100% (*p* < 0.001), while in the non-ELF-EMF group, it did not change (*p* > 0.05) (Figure 2).

**Figure 3:**
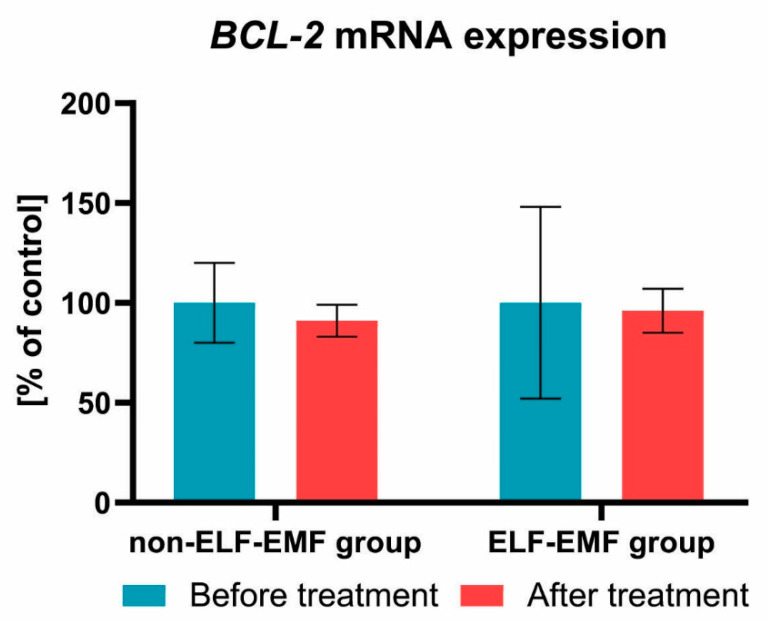
Comparison of the *BCL-2* mRNA expression acquired from the study group vs. control. Results formulated as parameter changes before and after therapy (100% expressed for the level of *BCL-2* mRNA expression in each patient sample before treatment).

**Figure 4:**
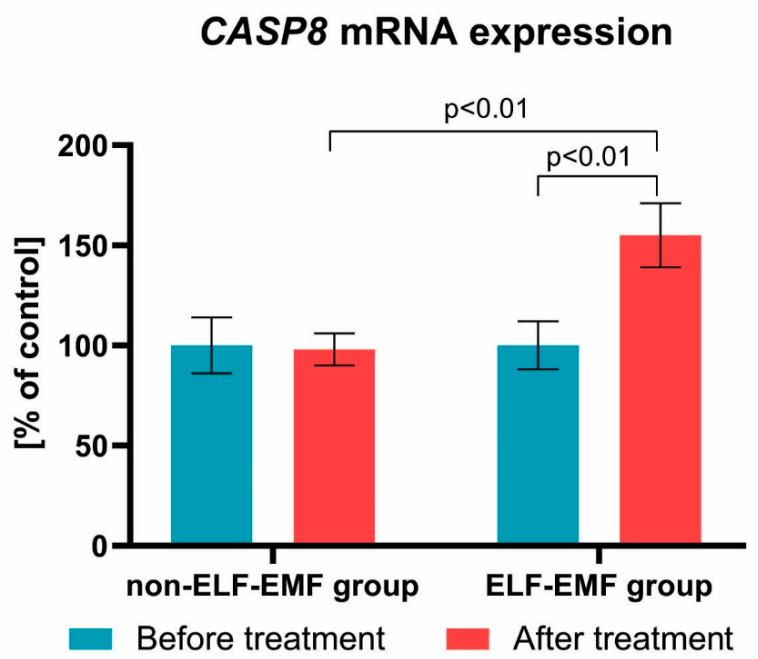
Comparison of the *CASP8* mRNA expression acquired from the study group vs. control group. Results formulated as parameter changes before and after therapy (100% expressed for the level of *CASP8* mRNA expression in each patient sample before treatment). Statistical significance between ELF-EMF and non-ELF-EMF groups after 10 sessions amount to *p* < 0.01.

**Figure 5:**
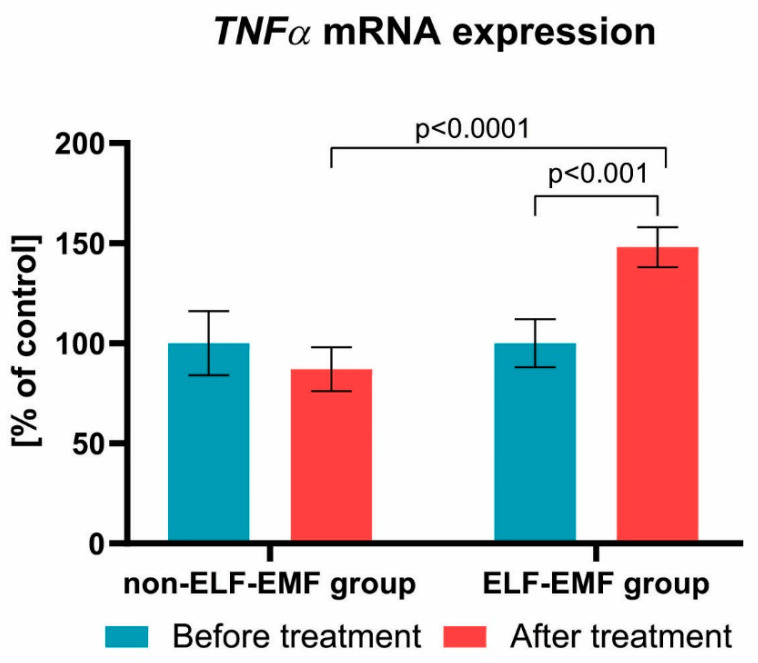
Comparison of the *TNFα* mRNA expression acquired from the study group vs. control group. Results formulated as parameter changes before and after therapy (100% expressed for the level of *TNFα* mRNA expression in each patient sample before treatment). Statistical significance between ELF-EMF and non-ELF-EMF groups after 10 sessions amount to *p* < 0.0001.

We also found that the expression of the *BCL-2* gene after a series of 10 physical treatments remained at a comparable level in both groups (Figure 3). Subsequently, we estimated the impact of the ELF-EMF on CASP8 gene expressions in the whole blood samples. We showed that, after ELF-EMF therapy, the expression of *CASP8* mRNA in the ELF-EMF group increased by about 50% (*p* < 0.01), but in the non-ELF-EMF group, it remained at the same level (*p* > 0.05) (Figure 4). Similarly, as shown in Figure 5, the expression level of *TNFα* was increased in the ELF-EMF group by about 50% (*p* < 0.001), whereas in the non-ELF-EMF group, it remained at the same level (*p* > 0.05).

**Figure 6:**
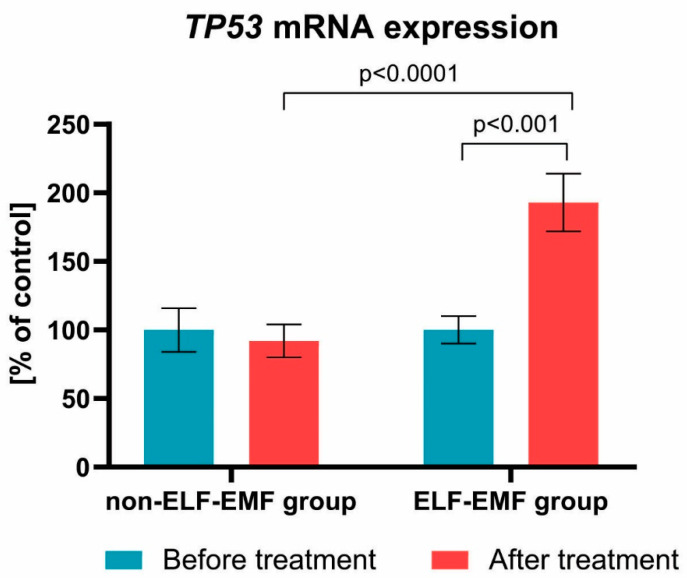
Comparison of the *TP53* mRNA expression acquired from the ELF-EMF group vs. the non-ELF-EMF group. Results formulated as parameter changes before and after therapy (100% expressed for the level of *TP53* mRNA expression in each patient sample before treatment). Statistical significance between ELF-EMF and non-ELF-EMF groups after 10 sessions amount to *p* < 0.0001.

Additionally, we examined the *TP53* mRNA expression level. We demonstrated that, after rehabilitation, the mRNA expression of *TP53* gene in the study group increased by about 100% (*p* < 0.001), while in the control group, it remained unchanged (*p* > 0.05) (Figure 6).

## 4. Discussion

An increased apoptotic process in acute phase of stroke is proven, and associated with the severity of neurological deficit [26,27]. There is still little information about apoptosis in the subacute/chronic phase of stroke. Within several minutes after ischaemia, neuronal cells in the infract core died [28], however, in penumbra, neuronal cells died slowly within a couple of hours after the incident [29]. Deng et al. studied the dynamics of apoptotic processes in mice that had experienced an induced stroke. They showed that markers of apoptosis had grown 2–5 h after ischemic, peaking after 5 h. At that time, the stroke volume and neurological deficit expended slowly, implying that apoptosis activation might inhibit ampliation of the ischeamic core and promote neuronal survival. Moreover, after this time, apoptosis was normalized [30].

Apoptosis, as programmed cell death, aims to eliminate damaged cells and, therefore, also those resulting from hypoxia/reperfusion. Magavi et al. investigated the neurogenesis of the adult cerebral cortex. They observed that induced apoptosis promoted the formation of new neurons [12]. Similarly, Chen et al. found the activation of neurogenesis in mice with induced apoptosis in corticospinal motor neurons [11].

Our innovative research concerned ELF-EMF activity in vivo in post-stroke patients. Most of the available studies were conducted on cell lines or animal models. The results obtained from research carried out using other research models cannot be precisely related to human rehabilitation. Thus, we relate current results only to our previous research that showed that ELF-EMF therapy improved neuroregeneration processes at the molecular level. In our previous papers, we found that rehabilitation treatments with the use of ELF-EMF intensification processes of neuroplasticity in post-acute stroke patients [21,22,23]. We found that, after exposition to ELF-EMF, the brain-derived neurotrophic factor (BDNF) expression at both the mRNA and protein level was increased, as well as plasma cytokine levels—hepatocyte growth factor (HGF), vascular endothelial growth factor (VEGF), stem cell factor (SCF), and interleukin 1β (IL-1β)—were elevated [22,25]. Moreover, we observed that ELF-EMF affected the synthesis of nitric oxide (NO), which participates in plasticity processes [23].

In this study, we demonstrated that ELF-EMF increased *BAX* mRNA expression in vivo in post-acute stroke patients (Figure 2). Our results concur with the research shown by Wang et al. [31]. They observed that 3 mT EMF promotes osteoclast apoptosis by up-regulation of *RANK*, *NFATc1*, *TRAP*, *CTSK*, *BAX*, and *BAX/BCL-2* expression, thereby enhancing osteoclast formation and maturation [31]. On the other hand, Tenuzzo et al. [32] assessed the effect of exposition of 6 mT EMF on expression of *BAX*, *p53*, *HSP70*, and *BCL*-*2* in human lymphocytes. They found that, after exposition, the *BAX* and *p53* gene expression rose, while the *hsp70* and *BCL*-2 gene expression decreased.

We observed that, after ELF-EMF rehabilitation, the mRNA expression of *BCL-2* remained at a comparable level in both groups. Our results concur with a study shown by Ding et al. [33]. They estimated the effect of ELF-EMF on H_2_O_2_-induced apoptosis and necrosis in human leukaemia HL-60 cells. They observed that the level of BCL-2 in H_2_O_2_-treated cells was comparable to its level in cells treated with both H_2_O_2_ and ELF-EMF. They also suggested that EFF-EMF itself cannot induce apoptosis and necrosis [33].

We demonstrated that the *CASP8* and *TNFα* mRNA expression were elevated in the ELF-EMF group (Figure 4; Figure 5). Xie et al. estimated the impact of EMF therapy on chondrocyte morphology and apoptosis, and the expression of apoptosis-related proteins in rabbits with anterior cruciate ligament transection. They found after, exposition on ELF-EMF, the expression level of *CASP8* was higher in comparison with the healthy group, but that there was an insignificant difference in comparison with the untreated group with anterior cruciate ligament transection. The EMF group was also characterized by better clinical parameters [34].

In our current study, we found that expression of *TP53* mRNA increased by about 100% (Figure 6) in the ELF-EMF group. Vincenzi et al. [35] evaluated the impact of pulsed electromagnetic fields (PEMF) on p53 activation and the stimulation of A3 adenosine receptors in NF-kB, cytotoxicity, apoptosis, and cell proliferation. They found that PEMF treatment alone was not enough to affect regulation of p53 expression. However, the coinstantaneous treatment of tumour cells with PEMF and agonist of A(3) adenosine receptors (A3AR)—2-chloro-N 6-(3-iodobenzyl)adenosine-5′-N-methyl-uronamide (Cl-IB-MECA)—caused a further significant elevation of protein levels of p53, compared with Cl-IB-MECA alone [35]. Similarly, Ma et al. [36] evaluated the impact of ELF-EMF on the proliferation and differentiation of neural stem cells. They observed that, after exposure to ELF-EMF, cell cycle analyses do not significantly change in the studied group compared with the control. Additionally, they found no significant difference in the expression level of the *p53* gene [36].

The potential mechanism of intensification of apoptosis processes by ELF-ELM could be related to its effect on calcium channels. The effect of ELF-EMF on Ca^2+^ flux is well documented [37,38,39,40]. The increase of Ca^2+^ ions is an alarm signal that may present differently according to the physiological details. It has been shown that apoptosis can be dependent on an increase in the level of mitochondrial calcium, which in turn induces the release of mitochondrial cytochrome c and activation of proteases [41]. In addition to intrinsic and extrinsic pathways of apoptosis, there are also reticular pathways. The reticulum pathway is caused by disturbance of the ion balance (especially Ca^2+^ ions), and the accumulation of improperly folded or modified proteins in the cell. In the caspase-independent pathway, calpain plays an important role in apoptosis. Calpain is a cysteine protease activated by Ca^2+^ ions. After stimulation, calcium is released from the endoplasmic reticulum and is bound with many factors, including with calpain [42].

Morabito et al. [43] evaluated the impact of ELF-EMF on morphology, proliferation, and differentiation in pheochromocytoma cells (PC12), as well as on the induction of oxidative stress dependent on Ca^2+^. Oxidative stress causes overproduction of reactive oxygen species (ROS), which leads to apoptotic cell death. Cell growth and viability were examined after ELF-EMF exposure (50 Hz, 01,1 mT) using morphological and colorimetric analysis. Assay immediately after exposure demonstrated an increased level of ROS and a decreased catalase (CAT) activity, without affecting the Ca^2+^ level. On the other hand, seven days’ exposure caused an increased CAT activity, which may suggest the cell adaptation on ELF-EMF. Furthermore, long-term exposure caused an intracellular Ca^2^ level. Calcium activates voltage-gated (L-type) Ca^2^ channels, which, via cell pathways (extracellular signal-regulated kinases, c-Jun N-terminal protein kinase/stress-activated protein kinase, and p38), could lead to the activation of gene expression regulating apoptosis, cell survival, and differentiation [43]. Our previous research is compatible with Morabito studies. We observed that, two weeks post-stroke, rehabilitation with ELF-EMF exposition caused an increase in antioxidant enzymes activity: CAT and superoxide dismutase (SOD) [24], as well as elevation of the expression of antioxidant enzymes genes: *CAT*, SOD (*SOD1* and *SOD2*), and glutathione peroxidase (*GPx1* and *GPx4*) [21]. Moreover, we demonstrated that 10 sessions of ELF-EMF therapy decreased the level of parameters of oxidative stress in patients after stroke [24].

What is particularly important in our previous works is that we reported that, after ELF-EMF treatment, the improvement of biochemical markers was accompanied by the enhancement of clinical parameters. ELF-EMF improves motor condition expressed in the ADL scale, as well as mental efficiency assessed in the MMSE and GDS scales [21,22,23,24,25]. Our research to date clearly shows that ELF-EMF significantly boosts the effectiveness of rehabilitation. In our current study, we established that ELF-EMF additionally increases the expression of pro-apoptotic genes. Enhancement of apoptosis in post-stroke rehabilitated patients can significantly contribute to improving repair processes and increasing neuroplasticity owing to the removal of redundant or damaged cells.

On the basis of all of our research, we suggest that increasing the expression of these genes in actively rehabilitated post-stroke patients promotes the induction and/or intensification of signaling pathways involved in brain plasticity. However, subsequent study is needed to elucidate the exact mechanism of this process, which can include a simultaneous action of a variety of repair systems. Nevertheless, we recommended that the inclusion of ELF-EMF therapy could intensify the efficacy of the treatment after stroke.
